# Women in chemistry: Q&A with Professor Nicola Gaston

**DOI:** 10.1038/s42004-026-01995-8

**Published:** 2026-04-09

## Abstract

Professor Nicola Gaston is the Director of the MacDiarmid Institute for Advanced Materials and Nanotechnology, a New Zealand Centre of Research Excellence, and a Professor in Physics at the University of Auckland, where her research group conducts quantum-mechanical simulations of the electronic and thermodynamic behaviour of nanoscale materials.

**Figure:**
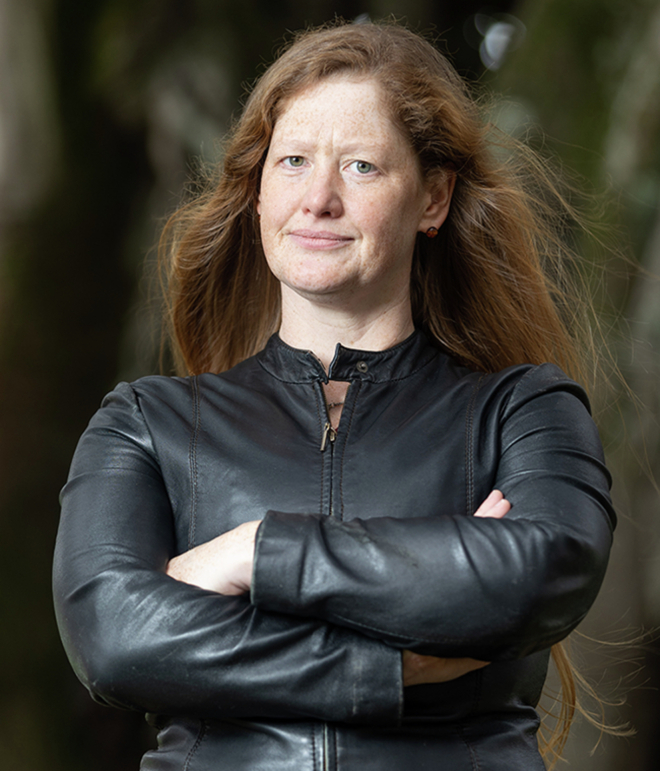
Elise Manahan for University of Auckland

**Nicola’s goal is to find new ways of making materials that address issues of sustainability—whether addressing issues of energy sustainability, or by designing materials to be more sustainably synthesised and indeed recycled**.

She was elected a Fellow of the Royal Society Te Apārangi in 2023 for her important insights into the behaviour of low-temperature liquid metals. That same year, she was awarded the Thomson Medal of the Royal Society Te Apārangi for transformative leadership for the research, science and innovation sector. She also served as the elected President of the New Zealand Association of Scientists in 2014 and 2015.

Why did you choose to be a scientist?

I have to answer this first with an extended ‘hmmmm’. I don’t know that I did! Or at least, I would say that it was never a single decision but a set of them, over an extended period of time: first, the recognition that I enjoyed maths at school; later on, realising that both physics and chemistry really interested me. The school system tended to encourage us towards medical training if you were good at science. I did not find that appealing, and to be fair I had had very little interaction with doctors at that point in my life—the only thing I remember my mother taking us to see our family doctor for was prescription sunscreen! Engineering was the other somehow prestigious science-based career from my teachers’ perspectives (on account of being a restricted entry university programme), but my father was an engineer, which ruled that out quite decisively. In the end, I am a scientist because I went to university to learn, and the most important thing I learnt is that research is how we learn things beyond what is already known. So, somehow, I have managed to never stop.

Have you been a minority as a woman at any stage of your career? What was that experience like for you?

I travelled back to Dresden a couple of years ago for the 30th anniversary of the Max Planck Society in Saxony, and walked into the meeting room of the Max Planck Institute for the Physics of Complex systems, where I was a postdoc two decades ago. I was a little late, so I stood at the back and it took me a while to take in the room—maybe somewhere over a hundred people, and my best estimate is that we were 2 women in the room. I had really forgotten what that felt like—it has not been my experience for a decade or more—and it really took me back.

As an undergraduate student in first-year physics, I do remember having been given the full metal jacket treatment—the ‘look to your right, now look to your left: one of you will fail this course’. I remember finding it rather cringeworthy at the time, but even more than that, I remember it concentrating my attention on who was in the class of a few hundred students—success rates in undergraduate physics may be a matter of statistics, but women were most naturally counted in units of the fingers on your hand.

I’ve had the extraordinary privilege in my career to have worked in many places and many environments, which has allowed me to add context to these experiences. I’ve worked in both universities and in public research institutions; in New Zealand and in Europe. From a PhD in theoretical chemistry, I moved to physics, then to an applied mathematics group, then back to chemistry, and then again back to physics. Even just the first few of those moves gave me a strong impression of just how much the experience of being a woman in science is context-dependent—and yes, the most important part of that context is how many other women are in the room. But there are male-dominated environments and workplaces in which you can absolutely sense that the direction of change is positive—where that is the case, it is not always a necessarily negative experience.

What scientific development are you currently most excited about?

Currently, I have to vote for what we are discovering—from the most fundamental aspects to a very wide range of technological applications—about the behaviour of low temperature liquid metals, such as gallium. I have always been fascinated by the simple fact that while the majority of elements in the periodic table are metals, only very few of them are liquid under anything close to standard conditions. And, of course, there is a version of the anthropic principle involved—standard conditions are determined by the size of our planet and our distance from the Sun—but that their behaviour is so different from what we think of as normal for a metal is nonetheless striking.

It’s a particularly nice playground for theoretical and computational work, since the question I find most satisfying to answer as a scientist is ‘why?’. So being able to note that mercury melts at a low temperature on account of relativity, but that gallium melts at a low temperature on account of its ability to have covalent and metallic bonds coexist, is really satisfying. Even more so, our recent realisation that so much confusion in the literature regarding the structure of liquid gallium could be reconciled by the recognition that covalent bonds in the liquid metal only exist at higher temperature, and not in the liquid metal upon melting, was a real delight. Gallium still has a low melting temperature on account of having a covalent or dimeric character in the solid—but we can now say that it is not because it melts as a molecular solid, requiring less enthalpy than normal to drive the transition. Instead, it seems to be entropically driven, as the entropy difference between the solid and liquid is anomalously large as it melts from a dimeric solid to a monomeric liquid. I just think this kind of result—entirely unexpected—is cool.

What direction do you think your research field should go in?

This is an interesting one. I will start by saying that I am a firm believer in the importance of fundamental, or curiosity-driven or blue skies science—the idea that the expertise of the individual scientist should guide the choice of research topic and research pathway. Too often, funding and career pressures push scientists into ‘hot topic’ areas, leading to, in my opinion, a relative waste of energy and effort as too many individuals rediscover the same roadblocks or discover in parallel the same incremental results. This is not a criticism of individuals; it is a problem of how we fund science. I would love for us to be better about supporting scientists to diversify their efforts and to explore relatively understudied areas.

At the same time, there are some critical issues facing humanity, in particular around energy and material sustainability, and these are issues to which materials science and chemistry are central. So I do think we should use our values, our interests, and our ambitions for the world we live in to guide our scientific choices. More and more, I have come to believe that there is a false dichotomy between fundamental and applied science in this context. We should talk about fundamental *questions*; where is the key knowledge gap, the unknown, the conceptual advance that can shift the whole field? If those questions are relevant to a technological application—even if they are motivated by very directed or even commercially-focused research—that does not make them less fundamental. The danger is in a scientific model that says that working in an application *area*—let’s say solar energy—is important because that area of research is important. That is the trap, and it is one that citation metrics have too often led us into. We need to be better about assessing research questions, and the impact of their answers.

With that lengthy preamble out of the way, one of the biggest issues that motivates me, right now, is the issue of critical minerals. This is only in part because I am annoyed by having read far too many articles that discuss the ‘rare earths’ and include cobalt and lithium among them! But it is clear that there is an international conversation going on about the geopolitics of critical minerals—their mining, processing, and geopolitical availability—and chemists and materials scientists absolutely need to be weighing in on this. We have for decades been looking for alternatives to scarce, expensive or toxic elements using chemical intuition and materials design—and with the advent of AI and quantum computation for materials discovery on the horizon, this process will only get faster, cheaper, and more disruptive. So I’d like us to be focused on reframing what politicians only see as a geopolitical conversation into a scientific one, and I think there is a real need for this to be done with some haste.

How would you describe your research philosophy?

Very simple, really. The job is to discover things. Knowledge. Application of that knowledge as well. Perhaps the only way in which it gets complicated is when you ask ‘how’ we should do research; I do think that is a harder question.

If we are primarily guided by curiosity, and the desire for new knowledge, then I think that suggests the responsibility to keep an open mind about where that knowledge comes from. We should be willing to be educated by our peers, and to understand the process of research—peer review, and the need for reproducibility—as requiring teamwork. We must be willing to be wrong, and to acknowledge it when we are. We must be willing to learn from our students! And this I believe quite strongly, in fact: that while research-led teaching is a key responsibility of a university, teaching-led research is one of the fundamental benefits of an academic job to the academic.

Has geographical location or specific institute membership played a role in your experience as a woman in chemistry?

Oh, absolutely. Returning to explore a research career in New Zealand in 2007 required me to be flexible about where I wanted to go, career-wise—it is a small country, in particular so far as the science system is concerned—and this required the shift from a physics department, to applied maths, to chemistry, to physics (as I mentioned earlier), and the corresponding move from a research institute to a university environment.

I actually think that that disciplinary flexibility—almost an agnosticism with respect to my scientific identity—has made one of the most rewarding contributions to my career. Research is about learning, and sidewise shifts—perhaps not dramatic ones, but just enough to find myself challenged on jargon—have been very good for me. Perhaps less expectedly, it has made a deep impression on my experience as a woman in science. The idea of a scientific identity seems to be tied up quite intimately with both unconscious biases—who sees themselves as belonging in science—and the conscious expressions of those biases.

An unexpected privilege of being a woman in science is that I do not see being a scientist as my primary identity, and sometimes I would even like to imagine myself doing something else—if only there were not so many scientific questions still to be answered! But I definitely see a form of scientific identitarianism as the other extreme from my experience, and a sad one. If science is a human pursuit it must be a collective one. Those who make it about themselves, and about their own ego, are doomed to disappointment. And I am afraid that generations of men have been socialised into precisely this: spam emails and even physical mail from gentlemen who claim to have found a ’theory of everything’ are not a mystery to anyone who works in a physics department, but they are just the tip of the iceberg. I hope that we can provide a healthier perspective to future generations of young people with an interest in scientific questions.

Could you describe a memorable moment in your career where being a woman made a significant difference, possibly even steered your path in a certain direction?

In 2015 I published a short book called ‘Why Science Is Sexist’. I was perhaps naïve, but I felt able to write about the extensive literature on unconscious bias from the perspective of a woman in a male-dominated area of science and ask the question: why is science so biased (when viewed from the objective metric of relative participation of women and men) when my experience of working in science had generally suggested that my male colleagues were equally interested in issues of fairness and equity?

I have joked about the fact that presenting on the topic after the book was published was a form of crowd-sourced therapy; it was genuinely rewarding, affirming, and has led to a number of wonderful relationships and firm friendships that I would never otherwise have had. However, what I honestly did not expect was the reaction from men who saw themselves as progressive but nonetheless found themselves challenged by the fact that I wrote such a book. That definitely opened my eyes as to how complex the issue is. The biggest difference, however, is that writing the book changed how people saw me. In a professional meeting, I would suddenly have a senior male colleague look at me, and then raise the issue of equity or diversity with the group. Suddenly, I didn’t have to do quite so much work—others would do it for me.

I have often thought about the things I would say differently, a decade later, on the topic. There are certainly a host of issues that change with career stage, but even more, writing the book put me on a pathway to experiences that I would not otherwise have had. But I can honestly say I have never regretted publishing it. I would even update it, but unfortunately, I can’t quite say if the last decade has put it out of date, or made the issues it discusses even more relevant! Perhaps one day I will decide…

*This interview was conducted by the editors of Communications Chemistry*.

